# The microbial dark matter and “wanted list” in worldwide wastewater treatment plants

**DOI:** 10.1186/s40168-023-01503-3

**Published:** 2023-03-28

**Authors:** Yulin Zhang, Yulin Wang, Mingxi Tang, Jizhong Zhou, Tong Zhang

**Affiliations:** 1 Environmental Microbiome Engineering and Biotechnology Lab, Department of Civil Engineering, The University of Hong Kong, Pokfulam Road, Hong Kong, China; 2 Institute for Environmental Genomics, Department of Microbiology and Plant Biology, and School of Civil Engineering and Environmental Sciences, University of Oklahoma, Norman, OK USA; 3 Shenzhen Bay Laboratory, Shenzhen, China; 4 Peking University Shenzhen Graduate School, Shenzhen, China; 5 Macau Institute for Applied Research in Medicine and Health, Macau University of Science and Technology, Macau, China

**Keywords:** Wastewater treatment plants, Activated sludge, Biofilm, Anaerobic digestion sludge, Microbiome, Microbial dark matter, Genome, Wanted list

## Abstract

**Background:**

Wastewater treatment plants (WWTPs) are one of the largest biotechnology applications in the world and are of critical importance to modern urban societies. An accurate evaluation of the microbial dark matter (MDM, microorganisms whose genomes remain uncharacterized) proportions in WWTPs is of great value, while there is no such research yet. This study conducted a global meta-analysis of MDM in WWTPs with 317,542 prokaryotic genomes from the Genome Taxonomy Database and proposed a “wanted list” for priority targets in further investigations of activated sludge.

**Results:**

Compared with the Earth Microbiome Project data, WWTPs had relatively lower genome-sequenced proportions of prokaryotes than other ecosystems, such as the animal related environments. Analysis showed that the median proportions of the genome-sequenced cells and taxa (100% identity and 100% coverage in 16S rRNA gene region) in WWTPs reached 56.3% and 34.5% for activated sludge, 48.6% and 28.5% for aerobic biofilm, and 48.3% and 28.5% for anaerobic digestion sludge, respectively. This result meant MDM had high proportions in WWTPs. Besides, all of the samples were occupied by a few predominant taxa, and the majority of the sequenced genomes were from pure cultures. The global-scale “wanted list” for activated sludge contained four phyla that have few representatives and 71 operational taxonomic units with the majority of them having no genome or isolate yet. Finally, several genome mining methods were verified to successfully recover genomes from activated sludge such as hybrid assembly of the second- and third-generation sequencing.

**Conclusions:**

This work elucidated the proportion of MDM in WWTPs, defined the “wanted list” of activated sludge for future investigations, and certified potential genome recovery methods. The proposed methodology of this study can be applied to other ecosystems and improve understanding of ecosystem structure across diverse habitats.

Video Abstract

**Supplementary Information:**

The online version contains supplementary material available at 10.1186/s40168-023-01503-3.

## Introduction

Wastewater treatment plants (WWTPs) are one of the largest applications of biotechnology in the world [1], which utilize microorganisms to remove pollutants. As a typically engineered ecosystem with important functions, WWTPs harbor a large number of microbial populations and extremely high diversities of community structure [2–4] that are of great value to both engineering and fundamental research [5]. And knowing the global diversity and number of microorganisms in WWTPs is crucial for understanding the structure of this ecosystem. As the most commonly used taxonomic and phylogenetic identifiers for bacteria and archaea, the sequences of the 16S rRNA gene have enabled a detailed and fine-grained expanded understanding of prokaryotic communities, distribution, and dynamics in various ecosystems on both spatial and temporal dimensions [6–8]. In 2010, scientists all around the world cooperated to promote the Earth Microbiome Project (EMP) [9, 10] to characterize global microbial taxonomic and functional diversity on Earth *via* 16S rRNA gene. This project has greatly promoted the taxonomy and distribution of microorganism research in various ecosystems including WWTPs. For instance, Ju and Zhang [3] detected the temporal dynamics of bacterial communities in Hong Kong activated sludge (AS) samples for five years. Matar et al. [11] tried to define core bacterial community in full-scale membrane bioreactors with 16S rRNA gene. And in 2019, a worldwide survey of 269 WWTPs in 23 countries based on 16S rRNA gene [12] estimated that AS contained a total number of 4 – 6 × 10^23^ bacteria and 2.0 ± 0.2 × 10^9^ species as an important pool in the whole Earth microbiome (~ 10^12^) [13].

In recent years, high-quality and complete genome sequence-based analyses begin to take dominant positions in research due to the rapid development of sequencing technology and the diversity of research objectives. Compared to the low phylogenetic resolution and primer mismatches of 16S rRNA gene amplicon sequencing [14], high-quality and complete genome sequencing could offer a finer level of genomic features to satisfy metabolic research and other further exploration. Besides, the genome provides a layout for the evolution and function of prokaryotes with genetic information and improves our understanding of their interactions with the surroundings [15]. Cultivation is one of the most important microbiological research methods which not only can get pure cultures for subsequent biochemical analysis but also may obtain corresponding complete genomes through sequencing. The paradigm that only 1% of microbes are culturable has had a profound impact on our understanding of microbial ecology [16, 17], and the majority of prokaryotes on Earth have not been isolated and sequenced [18]. These lineages may have physiologies that prevent growth in pure culture in the lab due to various reasons, such as precise chemical or physical parameters that are difficult to maintain [19], extreme dependence on oligotrophy [20–22], or very low growth rates [23]. In recent years, cultivation-independent methods, such as metagenomics, single-cell sequencing [24], and hybrid assembly [25, 26] of the second- and third-generation sequencing, overcome the bottlenecks of cultivation and show enormous potential in genome mining. For example, Wang et al. [27] recovered 920 metagenome-assembled genomes (MAGs) of prokaryotes from a full-scale WWTP with the sequencing data over 9 years to study the successional dynamics of the community. Up to now, more than 300,000 genomes have been sequenced and deposited in public databases, such as the Genome Taxonomy Database (GTDB) [28], which constructs the phylogeny with the genomes obtained from RefSeq and Genbank of the National Center for Biotechnology Information (NCBI) database after quality control.

Although prokaryotes comprise the majority of the WWTPs’ biomass, we are still short of knowledge on their roles in ecosystems because most prokaryotes and their genomes remain uncharacterized [29]. These unknown microbes are colloquially called “microbial dark matter” (MDM) [30]. Understanding the abundance of MDM in environments is important to the field of microbiology as microbe-dominated ecosystems cannot be reliably characterized without a thorough understanding of MDM’s roles in ecosystem processes. The current knowledge of microbes mainly comes from a few isolated/sequenced taxa and the uncultured/unsequenced majority presumably constitutes important evolutionary [31]. For example, two new superphyla with novel metabolic traits were defined by uncovering genomic data of MDM to deepen our understanding of phylum-level relationships of the tree of life [32]. Combining the 16S rRNA gene and genome information, a recent study [33] conducted a large-scale sequence alignment between EMP data [10] and the genomes in the public database, and approximated the median proportions of the genome-sequenced cells and taxa (at 100% identities in V4 region of 16S rRNA gene) in different environments reached 38.1% and 18.8%. Yet, this study did not include an in-depth discussion on WWTPs. Although the global survey of prokaryotic diversity on WWTPs has been done [12, 34], there is no research so far providing that can clarify the proportions of MDM in WWTPs.

Here, we made a worldwide survey to elucidate the proportions of MDM in WWTPs by linking 16S rRNA gene amplicons and genomes, then defined the priority of these unknown prokaryotes, and finally proposed diverse methods to recover more genomes. Three types of important samples in WWTPs were chosen, i.e., AS for the major aerobic treatment process, aerobic biofilm as other types of wastewater treatment reactors like moving bed biofilm reactor (MBBR), and anaerobic digestion sludge (DS) for sludge treatment in WWTPs. The data was collected from the public database, including 1984 AS samples from 28 countries, 195 aerobic biofilm samples from 6 countries, and 854 anaerobic DS samples from 10 countries. To our knowledge, this is the first research to evaluate the present situation of MDM in WWTPs with the following three questions be answered: (1) what are the genome-sequenced proportions of prokaryotes in different processes of WWTPs; (2) what is the “wanted list” in AS of WWTPs; and (3) are there any other methods that can recover more genomes in WWTPs to help us clarify the system? It is worth noting that the methodology developed in this study is feasible and transferable to enable a more comprehensive understanding of other ecosystems.

## Methods

### Data collection of EMP and worldwide WWTPs

**Fig. 1:**
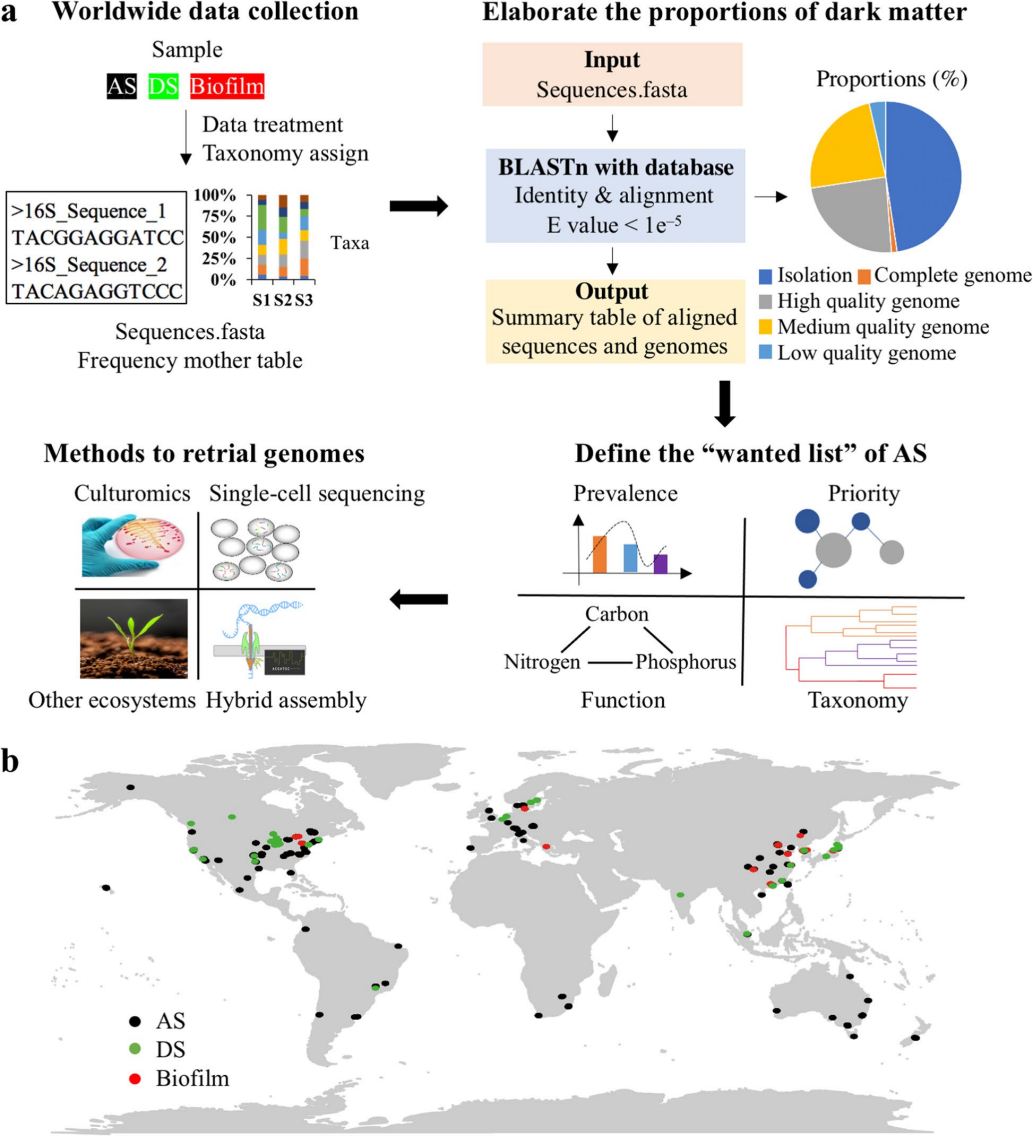
Overview of the analysis flows and sampling sites. **a** The flowchart of whole analysis steps. Firstly, the worldwide sequencing data was collected from public database and treated to generate sequences and mother table of frequency; secondly, the proportions of dark matter were elaborated with genome database; thirdly, the “wanted list” of AS was defined; finally, methods that could recover more genomes from WWTPs were discussed. **b** The sampling locations of worldwide WWTPs data. Three different systems are included: AS (dark), biofilm (red), and DS (green)

The EMP sampled the Earth’s microbial communities to advance our understanding of the microbial community structure on Earth [9, 10]. To compare the genome sequenced proportion of WWTPs with samples from the different environments on Earth, we downloaded the result table of 16S rRNA gene amplicon studies generated by EMP (ftp://ftp.microbio.me/emp/release1/otu_tables/deblur/emp_deblur_90bp.subset_10k.rare_5000.biom), which collected a total of 262,011 sequences in 10,000 samples taken from 17 microbial environments (EMPO level 3). The raw Illumina amplicons sequencing data for any regions of 16S rRNA gene in worldwide WWTPs were collected from the NCBI public database with the keywords of “activated sludge”, “biofilm”, and “digestion sludge”. Finally, we got 1984 AS samples in 28 countries, 195 aerobic biofilm samples in 6 countries, and 854 anaerobic DS samples in 10 countries (Fig. 1a) after the data quality check of the sequencing platform (Illumina) and quality (Phred quality > 19). Their NCBI accession number and other details are shown in Table S1. To avoid the potential deviation that might be caused by analysis methods, the data of WWTPs underwent the same analysis methods as EMP data, i.e., the single-end data of forward reads were trimmed to 90 bp and denoised by Deblur [35] in QIIME2 (v 2020.02) [36] to generate the amplicon sequence variants (ASVs) after quality filtration (Phred quality threshold of 19) and chimera removal by UCHIME2 [37]. Then, all samples were rarified to 20,000 reads to calculate the values of alpha diversity indices. The correlations between the genome-sequenced proportion and alpha diversity indices were measured by the Pearson correlation coefficient. The taxonomy of sequences was determined by Silva 138 database [38] with QIIME 2’s q2-feature-classifier [39].

### Subgroup classification of GTDB genomes

We downloaded 317,542 genomes (genomic_files_all) in GTDB (Release 207 version) [40] in July 2022 from the website (https://data.ace.uq.edu.au/public/gtdb/data/releases/release207/207.0/). According to GTDB metadata, the genomes were divided into five subgroups, i.e. “isolation”, “complete genome”, “high-quality genome”, “medium-quality genome”, and “low-quality genome”, respectively. We firstly consulted the “ncbi_genome_category” field in the GTDB metadata to establish if NCBI (as parsed by the GTDB) indicated a genome was derived from an environmental source or not and the value of “none” means the genome comes from isolates. Then the complete genomes were extracted from the column of “ncbi_assembly_level” with the keyword “Complete genome”. Finally, we collected the high-quality (HQ), medium-quality (MQ), and low-quality (LQ) genomes based on the value of the columns “mimag_high_quality”, “mimag_medium_quality”, and “mimag_low_quality”, respectively. Besides, 3533 genomes in three recent wastewater studies were also downloaded for analysis [27, 41, 42]. The genome quality was checked by checkM (v 1.0.7) [43] with the function of “lineage_wf” according to GTDB standard [44].

### Sequence alignment and analysis

Alignment between the amplicons and genomes was performed using BLASTn (default settings, *E* value < 1e^–5^) [45] with the amplicons as the query. The results matching 100% coverage and 100% identity were used to calculate the genome-sequenced proportions. We also calculated and summarized the results of 98.7% and 97% identity in the Supplementary information (Figure S4). The 100% identity represents the most rigorous and accurate match, while the results of 98.7% and 97% identities that represented the new and traditional criteria for species definitions were also summarized in the Supplementary Information. *P*_number_ and *P*_abundance_ were used to represent the genome-sequenced proportions of taxa (total ASV number) number and corresponding cell number (total relative abundance sum) sum in a specific environment. The whole flowchart was shown in Fig. 1b.

### The global-scale “wanted list” strategy for AS

For the global-scale “wanted list” analysis of AS, ASVs in V4 region (1208 out of 1984 AS samples) were extracted and clustered at a 97% level with vsearch (version 2.18.0) [46] to finally generate a mother table with 16,967 operational taxonomic units (OTUs). These OTUs could provide taxa identification at the genus level or above. A global-scale “wanted list” microbial community could be determined based on different criteria. In this work, we generated the “wanted list” from four aspects: prevalence, priority, function, and phyla with few representatives. The prevalence means OTUs should be universal (present in >40% samples) and have a higher relative abundance (> 0.1%, Table S2) [34], which is usually used to define “core microorganisms” in other studies. To meet multiple research objectives, we also defined the prevalence OTUs with tight (present in > 50% samples, Table S3) and loose (present in > 30% samples, Table S4) levels. Key species with high priority in the ecosystems that might be omitted only by the prevalence could be determined by the network analysis. Besides, the functional prokaryotes and phyla with few representatives should also be taken into consideration. These four criteria together generated the final global-scale “wanted list” for AS, including 71 OTUs and four phyla.

### Network creation, analysis, and priority definition

The network of priority criterion was constructed by the spiec.easi() function from the package SpiecEasi (version 1.1.1) [47] with the default settings of the “mb” parameter to estimate the conditional dependence of each pair of OTUs. To reduce sparsity and ensure robust results, only the OTUs presented in a given percentage (30%) of samples were reserved for network analysis. The parameters of within-module connectivity (Zi) and among-module connectivity (Pi) [48] were used to classify the nodes into four categories: module hubs (Zi > 2.5 and Pi < 0.62), connectors (Zi < 2.5 and Pi > 0.62), network hubs (Zi > 2.5 and Pi > 0.62), and peripherals (Zi < 2.5 and Pi < 0.62). Among these four categories, hubs (module hubs and network hubs) were typically the most connected taxa within the community and had the importance in an ecosystem network [31, 49]. Thus, the OTUs defined as hubs were included in the “wanted list” of AS. The network was visualized with the “igraph” package in R (version 4.1.0) [50].

### Abundance comparison of “wanted list” OTUs between WWTPs and EMP natural habitats

The 71 OTUs were aligned as query *via* BLASTn (*E* value < 1e^–5^) [45] with EMP sequences and then sequences meeting the standard of 100% alignment and 100% identity were picked. Once the sequences were confirmed, their highest abundance in AS and EMP samples and corresponding sample information were extracted from the mother table and summarized in Table S2.

## Results

### Low genome-sequenced proportions for prokaryotes in WWTPs

**Fig. 2:**
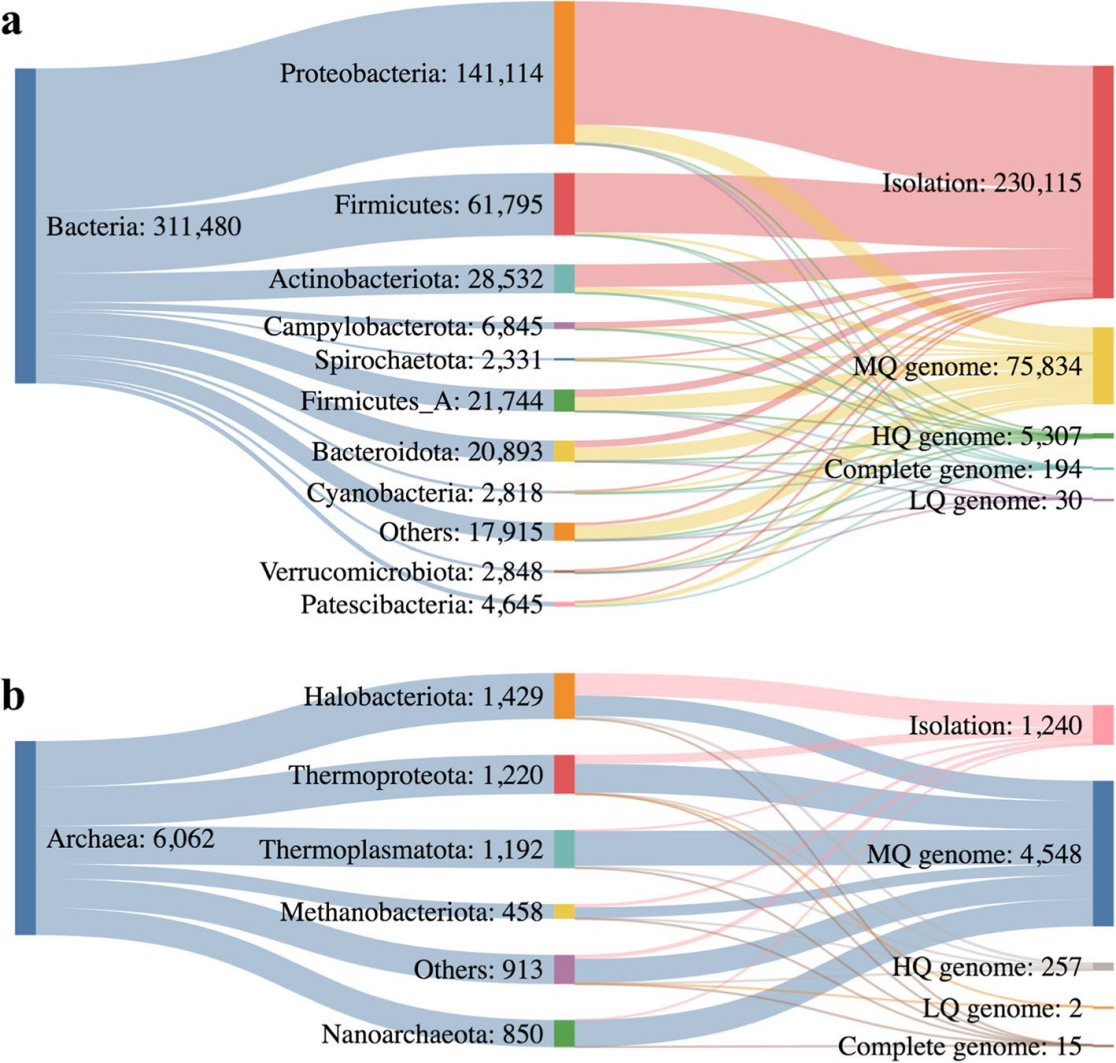
Basic taxonomy composition of GTDB R207, including **a** Bacteria and **b** Archaea. Only the top ten phyla of bacteria and top five phyla of archaea are shown in figure and the remaining phyla are merged as others. **a** and **b** have individual scale. The figures were made with SankeyMATIC. (HQ: high quality, MQ: medium quality, LQ: low quality)

The GTDB R207 contained 317,542 genomes of prokaryotes, including 311,480 bacteria and 6062 archaea, respectively. Figure 2a showed genomes of bacteria mainly came from 230,115 isolation strains (73.9%), and the remaining genomes were from as-yet uncultivated organisms (MAGs or single-amplified genomes (SAGs)), including 194 (0.1%) complete genomes, 5,307 (1.7%) HQ genomes, 75,834 (24.3%) MQ genomes, and 30 (0.01%) LQ genomes. These genomes were taxonomically affiliated within 148 phyla with the top three phyla of Proteobacteria (141,114, 45.3%), Firmicutes (61,795, 19.8%), and Actinobacteriota (28,532, 9.1%). Among them, 100 phyla whose percentage was < 0.05% were combined as “others” in Figure S1. For archaea, the 6062 genomes spread in 18 phyla. Most genomes were collected from MQ MAGs (4548, 75.0%) (Fig. 2b), then 1,240 isolation (20.5%), 15 complete genomes (0.2%), 257 HQ genomes (4.2%), and 2 LQ genomes (0.1%). The top three phyla were Halobacteriota (1429, 23.6%), Thermoplasmatota (1220, 20.1%), and Thermoproteota (1192, 19.7%).

**Fig. 3:**
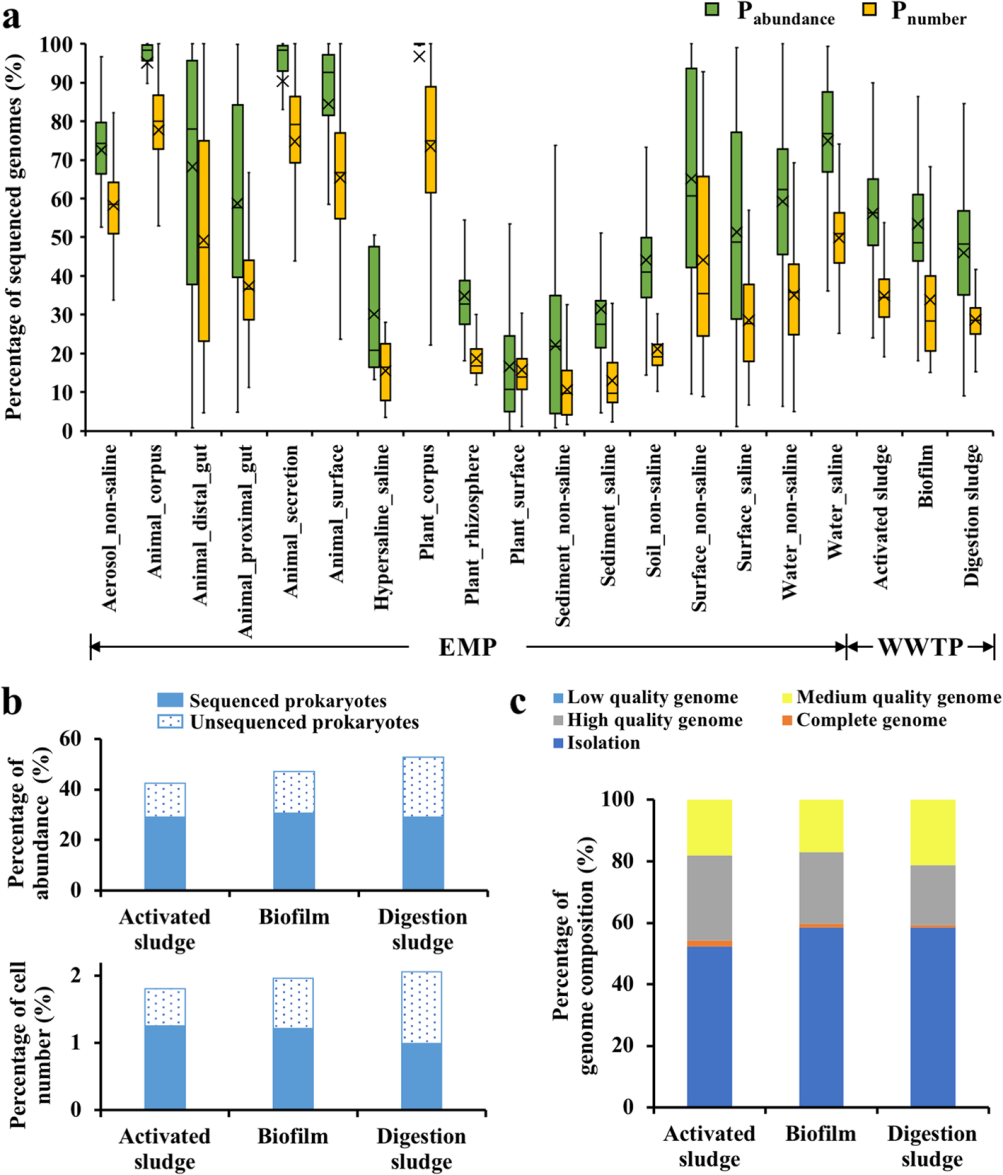
Genome sequenced results of environments in EMP and worldwide WWTPs. **a** Genomesequenced proportions of prokaryotes in different ecosystems. Yellow represents the genome-sequenced proportion of taxa (Pnumber) and green represents the sum of corresponding relative abundance (Pabundance) In specific ecosystems. OTUs share 100% identity and 100% alignment with the sequenced genomes. The results base on the analysis of 10,000 EMP samples with EMPO level 3 and the worldwide WWTPs samples. **b** The percentage of total cell number and abundance of top 1% taxa in activated sludge, biofilm, and anaerobic digestion sludge. The solid and dotted bar represents the percentage of sequenced and unsequenced prokaryotes, respectively. **c** Genome composition of sequenced prokaryotes. Blue, orange, grey, gold, and navy represent the genomes come from isolation, complete, HQ, MQ, and LQ, separately

After the establishment of genome categories, we analyzed the genome sequencing proportions of 10,000 EMP samples in 97 independent studies with 17 environment types (EMPO level 3). The results showed that the median of *P*_abundance_ in the 10,000 samples was 57.6% with the upper and lower quartiles of 31.7% and 91.3%, respectively (Fig. 3a). Besides, the median of *P*_number_ reached 34.8% (17.2–62.5%). These values increase obviously compared to another research with 38.1% for *P*_abundance_ and 18.8% for *P*_number_ [33] due to the rapid development of sequencing technologies in recent years. Among the diverse environmental types, the genome sequenced proportion of animal-related samples (93.3% of *P*_abundance_ and 68.2% of *P*_number_) was significantly higher than that of other groups (EMPO level 2, Figure S3). In detail, the median *P*_abundance_ of plant corpus (99.9%), animal corpus (98.3%), animal secretions (98.3%), and animal surface (92.6%) exceeded 90.0%, and their corresponding median *P*_number_ exceeded 50.0%. Of course, some environments might be really simple and only have a few taxa with the high abundance, such as plant corpus and animal corpus (Table S5). Comparatively, the median *P*_abundance_ values for plant surface (10.8%), hypersaline (20.8%), sediment_non-saline (21.9%), and sediment_saline (27.5%) samples were all less than 30.0%, and the median *P*_number_ values for sediment (non-saline and saline) samples were less than 10.0% (Fig. 3a). Despite significant differences, the genome sequenced proportions of prokaryotes were high in most EMP samples.

WWTPs had lower genome-sequenced proportions of prokaryotes (Fig. 3a) compared to EMP ecosystems based on the available data. The *P*_abundance_ and *P*_number_ of AS were 56.3% (47.9–65.1%) and 34.5% (29.4–39.2%). Similarly, the median *P*_abundance_ and *P*_number_ of biofilm were 48.6% (43.8%–60.9%) and 28.5% (20.7%–39.5%), respectively. For DS, medians of *P*_abundance_ and *P*_number_ were 48.3% (35.1–56.7%) and 28.5% (25.1–31.6%). From the WWTPs level, the *P*_abundance_ and *P*_number_ of all samples were 54.0% and 32.4%, which was much lower than animal samples and a little lower than Saline samples in EMP (Figure S3). When we released the identity criteria from 100% to 98.7% and 97%, the proportions of sequenced genomes increased but WWTPs still had almost one-third of unsequenced prokaryotes (Figure S4). These results mean the majority of the prokaryotes in WWTPs are uncharacterized. In fact, this result may still overestimate the real genome-sequenced proportions of the system. Because the alignment sequence length (90 bp) used in this research is short and some microbes may not be sequenced due to the limitation of sequencing technology or depth. Furthermore, we investigated the correlations between the genome-sequenced proportion and alpha diversity indices (observed OTUs, Shannon diversity, and Chao1) of EMP and WWTPs and found negative results, indicating samples with low alpha diversity tended to have a higher percentage of sequenced genomes (Figure S5). In summary, WWTPs had low genome-sequenced proportions of prokaryotes.

### The genome resolution of predominant prokaryotes in WWTPs is insufficient

We summarized the proportions of the total number and abundance of the taxa whose abundance was >1% in each sample and found that these small numbers of taxa contributed to a high relative abundance in different WWTP systems. According to Fig. 3b, these abundant taxa contributed to 42.3% relative abundance for AS, 47.0% for biofilm, and 52.8% for DS, while they only accounted for 1.8% cell number of AS, 2.0% of biofilm, and 2.1% of DS, respectively. By contrast, the rare taxa with low abundance (relative abundance of 16S rRNA gene < 0.1%) accounted for as high as 80.7%, 75.3%, and 73.3% of the total cell number of prokaryotes but only 18.0%, 10.0%, and 6.7% abundance for AS, biofilm, and DS samples, respectively (Figure S6). The same hyper-dominance patterns were observed not only in WWTPs [12] but also in other ecosystems [51]. For the prokaryotes with high abundance, it is important to get every single genome at least at the species level to help researchers explore communities. We further examined the resolution of these abundant prokaryotes (abundance > 1%) in WWTPs by calculating the percentage of the sequenced (solid bar in Fig. 3b) and unsequenced prokaryotes (dotted bar in Fig. 3b), respectively. Results showed that at least one-third of these prokaryotes were not sequenced, meaning the genome resolution of predominant prokaryotes in WWTPs is still insufficient.

Then we analyzed the genome composition of sequenced prokaryotes, and found around half of them were retrieved from pure cultures (52.2% for AS, 58.5% for biofilm, and 58.4% for DS in Fig. 3c). The remaining genomes mainly came from the HQ genome (27.6% for AS, 23.4% for biofilm, and 19.5% for DS) and MQ genome (18.1% for AS, 17.0% for biofilm, and 21.3% for DS). This phenomenon once again emphasizes the importance to dig out genetic information by sequencing techniques for current scientific research. In conclusion, the results suggest that the isolated strains are the main source of genomes currently, and culture-independent methods like metagenomics and single-cell sequencing also take a key position in genome mining of HQ and complete genomes.

### 71 OTUs and 4 phyla are defined in the global-scale “wanted list” of AS

**Fig. 4:**
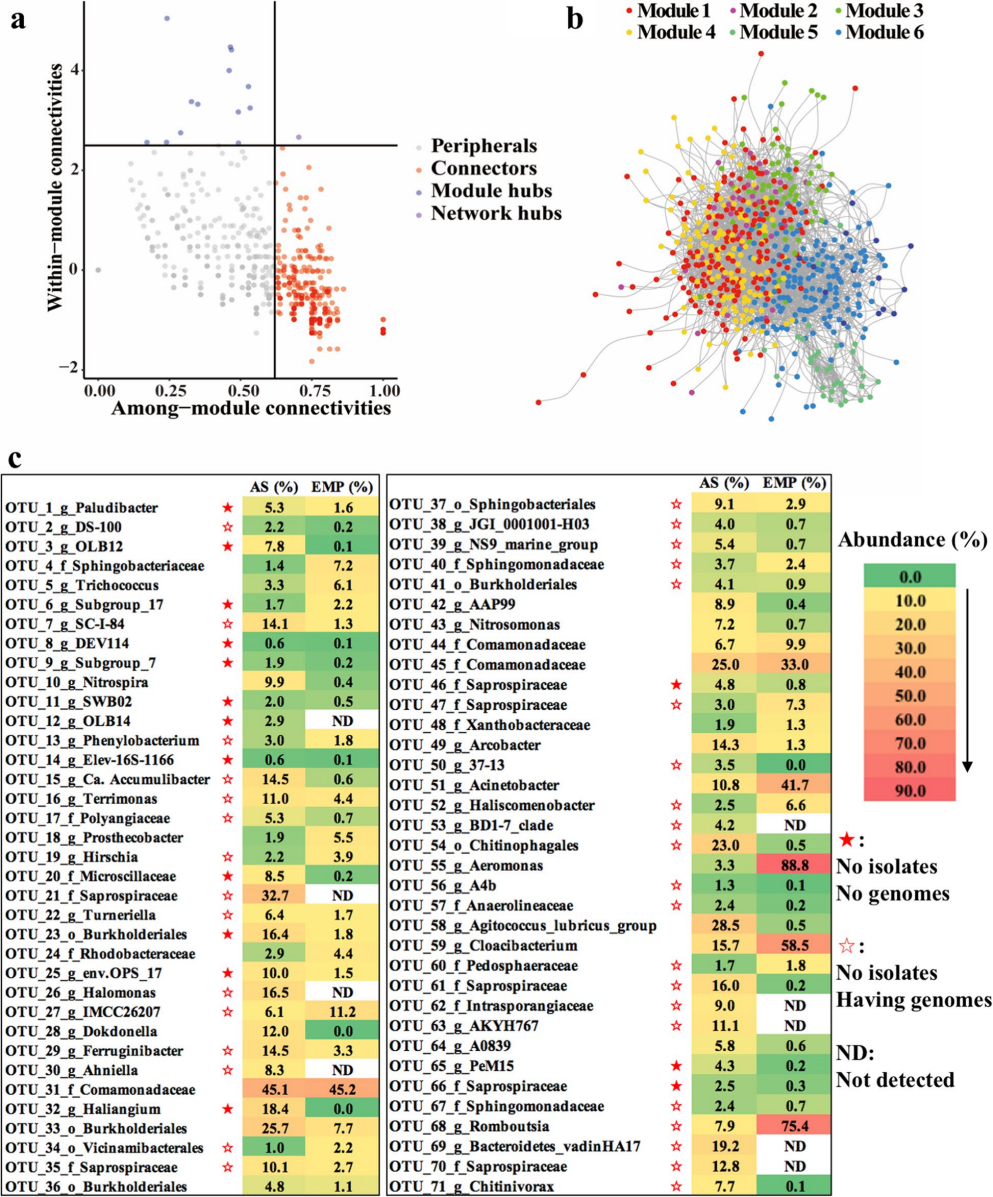
The network and “wanted list” of worldwide activated sludge. **a** The Zi (within-module connectivity) and Pi (among-module connectivity) scores of the worldwide activated sludge network. The nodes were classified into four categories, including module hubs (Zi > 2.5 and Pi < 0.62), connectors (Zi < 2.5 and Pi > 0.62), network hubs (Zi > 2.5 and Pi > 0.62), and peripherals (Zi < 2.5 and Pi < 0.62). **b** Overview of worldwide activated sludge network after removing low prevalence OTUs (show in < 30% samples) and independent nodes. **c** The “wanted list” of worldwide activated sludge. The 71 OTUs cover 39.7% averagely of total relative abundance in global activated sludge microbiota. The figure showed the highest abundance of those OTUs in EMP and activated sludge samples with 100% alignment and 100% identity

As the most representative process in WWTPs, AS was taken as an example to analyze the key species composition and generate the global-scale “wanted list”. The same approach is applicable to other ecosystems like biofilm and DS. We defined 61 OTUs with the prevalence standard that the OTUs should show in > 40% samples with a relative abundance > 0.1%. To meet multiple research objectives, we also defined the prevalent OTUs with loose (present in > 30% samples with a relative abundance > 0.1%) or tight (present in > 50% samples with a relative abundance > 0.1%) levels, and got 92 and 34 OTUs respectively (Table S3 and Table S4). Then, the network analysis was conducted to determine the notable species in AS. Here, 48.9% of the OTUs were peripherals with most of their links inside their modules (component units of the network), 48.8% were connectors (the species “glues” modules together), and 2.3% (14 OTUs) were hubs which are important to the network with supposedly notable ecology roles (Fig. 4a, b). Among the 14 OTUs, 12 OTUs had an average relative abundance lower than 0.1% in AS which meant their importance might be omitted by using the criteria of prevalence only. Combining the 61 prevalence and 14 priority OTUs (4 overlapping OTUs), we finally got 71 OTUs (Fig. 4C and Table S2). Besides, we summarized the top 10 phyla (67 phyla in total) in AS according to their abundance and identified four phyla having few representatives that should be taken into consideration, including the phyla of Patescibacteria, Planctomycetota, Verrucomicrobia, and Bdellovibrionota (Figure S7). Finally, the global-scale “wanted list” for AS was generated, including 71 OTUs covering the majority of the global AS microbiota (39.7% average of total relative abundance) and four phyla of few representatives.

It is of paramount value to know the dark part in the “wanted list”. The results showed only 20 OTUs had isolates, 36 OTUs had sequenced genomes, and 15 OTUs were poorly investigated without genome information (Fig. 4c, Table S2). No matter for genome mining or getting isolates for those unsequenced/uncultured OTUs in the “wanted list”, a good seed means a good start. Thus we searched the distribution of these 71 wanted OTUs in EMP samples (100% coverage and 100% identity) and found 62 OTUs presented with 18 OTUs had a higher abundance in EMP than AS samples (Fig. 4c), especially the OTU_55 (g_Aeromonas, had the highest abundance of 88.8% in Animal_distal_gut), OTU_59 (g_Cloacibacterium, had the highest abundance of 58.5% in Animal_corpus), and OTU_68 (g_Romboutsia, had the highest abundance of 75.4% in Animal_distal_gut). These results suggest that the gut microbes may migrate into WWTPs via influent and then grow or thrive in AS to play a role in the wastewater treatment process. This situation also hints us that species of some OTUs can be enriched start or even directly isolated from the corresponding EMP rather AS samples.

### The hybrid assembly method improves sequenced-genome proportions by capturing HQ and complete genomes

Connecting the 16S rRNA gene to the genome is limited due to the challenges of reconstructing HQ and complete genomes that included 16S rRNA genes by only short-read sequencing, especially for ecosystems with high complexity like AS [52]. However, the third-generation sequencing utilizes long reads to significantly enhance the contiguity of the assemblies or even recover complete genomes. And the hybrid assembly method that combined the long and short reads generated by the second- and third-generation sequencing technology, to a great extent, could resolve the limited recovery of 16S rRNA genes [53]. For example, Singleton et al recovered over 1000 HQ MAGs (including 57 closed circular genomes) from AS in Denmark using long-read and short-read sequencing [52]. Liu et al built a hierarchical clustering-based hybrid assembly pipeline and successfully recovered 557 MAGs with high contiguity from high-complexity AS samples in Hong Kong [42]. Notably, full-length 16S rRNA genes were identified in 410 MAGs.

**Fig. 5:**
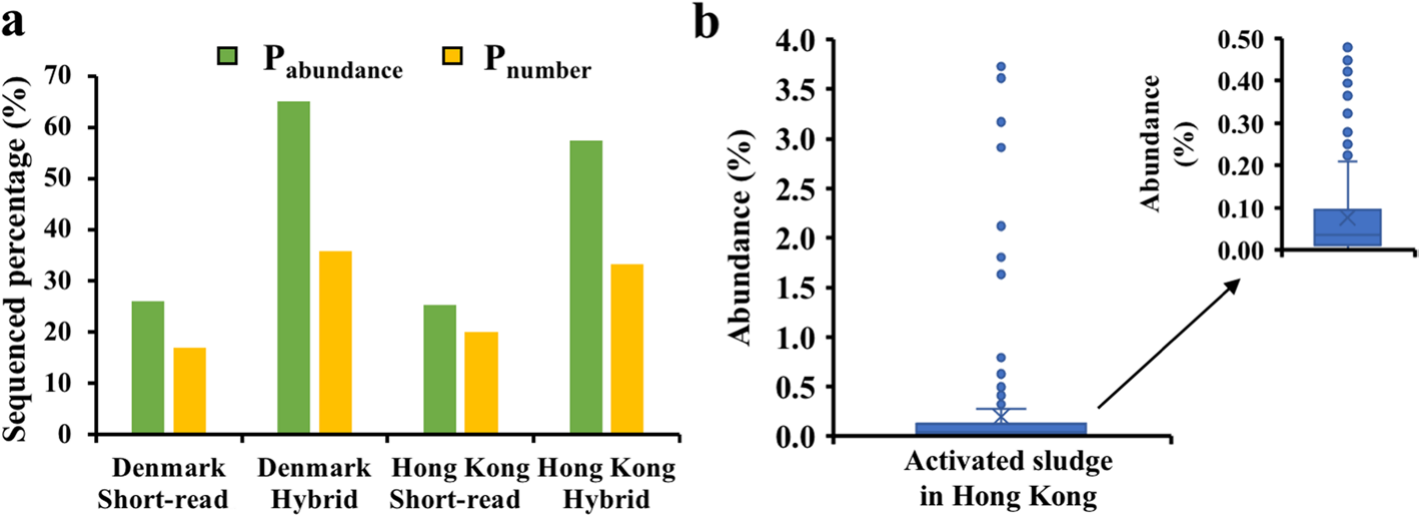
The results of hybrid assembly method. **a** The Pabundance and Pnumber of the sequenced genome before (Short-read) and after (Hybrid) adding the high-quality genomes in activated sludge of Denmark and Hong Kong. **b** The abundance distribution of recovered high-quality genomes in activated sludge of Hong Kong

To compare the contributions of short-read and hybrid assembly method to genome mining, we extracted the amplicons of AS in Denmark and Hong Kong from the collected dataset, recalculated the genome sequenced proportions by removing the hybrid assembly genomes from the database, and compared results with the values getting from the original database. According to the results in Fig. 5a, the median of *P*_number_ for Denmark AS decreased from 35.9 to 16.9% and the median of *P*_abundance_ declined obviously from 65.1% to 26.1% after removing the genomes recovered by the hybrid assembly method. For AS in Hong Kong, the *P*_number_ improved from 20.1 to 33.2% if the database included the hybrid genomes, and the *P*_abundance_ obviously increased from 25.3 to 57.5% due to the recovery of 20 high abundance genomes (abundance > 0.5% Fig. 5b) by the hybrid assembly method. This analysis indicated the distinguished application and huge potential of the third-generation sequencing in HQ and complete genome mining.

## Discussion

### The analysis between 16S rRNA gene and genomes reveals the high proportions of MDM in WWTPs

Genomes could give the blueprint of both taxonomy and diversity of complex microbial communities, provide important functions of microorganisms in metabolic processes, and reveal the potential roles of microorganisms in ecosystems. Although the sequencing batch effect of different studies may affect the results, medians of this work show prokaryotes in WWTPs have low genome-sequenced proportions, i.e., the high abundance of MDM. A study estimated that a large number of never-before-studied organisms might play important roles in ecosystem functions due to high levels of phylogenetic novelty [54]. From this perspective, our current knowledge of prokaryotic genomes in WWTPs remains limited because of the large number of unrevealed genomes in these huge genetic resources.

In fact, the real genetic resolution in WWTPs may be lower than our estimation even though we adopt strict criteria (100% identity and 100% coverage) because genomes contain much more genetic information and have more variants than the 16S rRNA gene [40]. Besides, the regions of 16S rRNA gene (like V4) only provide sufficient identification of taxa at the genus level or above, which is a historical compromise for the sequencing technology. For example, a study indicates that 56% of in-silico amplicons of the V4 region fail to confidently match their original taxonomic level [55]. However, the full-length 16S rRNA gene could provide taxonomic resolution of bacteria at species and strain level [56]. Today, the third-generation sequencing based on Pacific Biosciences [57] and Oxford Nanopore [58] platforms is capable of high-throughput sequencing of the full-length 16S rRNA gene with the high accuracy [59, 60]. What is more, methods used in this research only focus on chromosomes and could not reach to other genetic factors like plasmids, which play a distinct role in the process of the horizontal gene transfer of antibiotic resistance genes between bacteria [61]. In fact, plasmid sequences are difficult to recover by the current short-read sequencing since different plasmids often contain similar replications and conjugative elements [62, 63]. It also has difficulty assigning plasmids to corresponding MAGs [64]. Under such circumstances, the research of plasmids and other mobile genetic elements should be done to deepen our understanding of the MDM.

The ecosystems of WWTPs have high diversity and complexity with a few predominant species that contribute to high relative abundances. Predominant taxa always have higher priority for genome sequencing and isolation than taxa with low abundance and the above analysis shows that at least one-third of the predominant genomes have not been sequenced in WWTPs. This situation may be caused by several reasons. Firstly, the characterization of genomes mainly comes from pure cultures before the wide application of high-throughput sequencing, and the cultivation and genome research happens more often in the vicinity of humans [65] than environmental ecosystems, not to mention the artificial ecosystems of WWTPs. Secondly, the genome-sequenced prokaryotes with low abundance in WWTPs might originate and be characterized from other ecosystems. It is reasonable because WWTPs are open ecosystems where extraneous prokaryotes could be introduced through incoming raw sewage [66, 67], and the prokaryotes in WWTPs could also be easily spread to other environments *via* effluent discharge [68]. Additionally, WWTPs have their core bacterial community that shares no overlap with other ecosystems and needs more particular attention [12, 66]. Consequently, the representative prokaryotes in WWTPs still lack genome information, and thus more efforts should be paid to further explore this MDM for functional characterization to lay a solid foundation for subsequent system design, maintenance, and improvement. At the same time, the genome investigation of rare taxa is also important because they may have key functions in ecosystems [69, 70].

### The global-scale “wanted list” indicates the core microbes of AS need further research

The global-scale “wanted list” microbial community could be determined based on different criteria according to research aims. The most widely accepted and used criteria is to filter the taxa according to abundance (> 1% or 0.1%), such as 28 cores defined by a global bacterial study in WWTPs [12] (97% identity, 16S rRNA V4 region). Another study of global WWTPs further takes the conditionally rare or abundant taxa (CRAT) [34,71] into consideration and defined a core microbiota in AS with 966 genera. In this study, we combined the prevalence and priority (network defined) to eliminate the weakness of abundance that might neglect some key species having low relative abundance but potentially notable impacts on wastewater treatment and finally got a global-scale AS “wanted list” of 71 OTUs with the most of them belonging to Proteobacteria (38%) and Bacteroidota (31%) (Table S2). Although the “wanted list” obtained from different studies might have slight differences, the three studies all contain the typical taxa that are closely related to the performance of WWTPs, such as the genus of OLB12 (OTU_3) for organic matter degradation, Nitrospira (OTU_10) and Nitrosomonas (OTU_43) for nitrification, and *Candidatus* Accumulibacter (OTU_15) for phosphorus removal. Compared with the other two studies mentioned above, the “wanted list” in this research enriched the catalog through a network approach to explore the potential notable taxa such as the OTU_14 (g_Elev-16S-1166), which had the highest abundance of 0.6% in AS. OTU_14 still lacks genome/isolate information with no described species in this genus, and the in situ physiology of this genus in AS has not been determined.

The global-scale “wanted list” could facilitate various types of research in microbial ecology by specifying key targets. In 2020, a research team successfully isolated a new strain *Casimicrobium huifangae* which showed the ability to degrade complex organic matters [72] and belonged to 28 core OTUs mentioned above [12]. In our 71 “wanted list” OTUs, 15 OTUs are still in “darkness” with no isolates or genomes and 36 OTUs have genomes but lack isolates. This result verifies our previous conclusion that WWTPs still lack the genome information of representative strains, and once again emphasizes the importance of MDM studies in WWTPs.

Four phyla that are universal in AS but still have few representatives of genomes or isolates are also proposed to be paid more attention. Patescibacteria phylum [73] belongs to the *Candidate* phyla radiation group and widely exists in AS with high abundance. Planctomycetota and Verrucomicrobia phyla are the main members of the PVC group [74], which contains many interesting microorganisms with specific metabolic properties or important functions, such as anammox [75], a typical bacteria that could convert nitrite and ammonium ions directly into diatomic nitrogen and water but still no representative isolates. Bdellovibrionota phylum represents specialized bacterial predators that feed on a broad range of gram-negative bacteria and play a significant role in shaping the AS microbial community [76].

### Various approaches could be applied to elucidate genomes of MDM in WWTPs

A variety of approaches can be applied to further elucidate genomes of MDM in WWTPs. Currently, around half of sequenced genomes come from isolates. This result indicates that pure cultures still occupy an important position in the capture of genomes [77]. Besides, many novel technologies that provide complementary solutions for the shortcomings of traditional cultivation methods like low throughput have been spawned recently [56]. For example, the high-throughput culturomics method applies improved culture media to identify new roles of MDM [78]. In addition, microfluidics shows great potential in microbial studies because of the characteristics of automation and high efficiency [79]. Researchers designed the facile microfluidic streak plate to realize high-throughput single-bacteria cultivation of AS [80], and the single-cell sequencing based on microfluidics has been successfully applied to human gut microbiome [81] and should be utilized in AS. Besides, the next-generation physiology approaches that integrate techniques such as stable isotope probing [82], fluorescence in situ hybridization [83], confocal Raman microspectroscopy [84], and nano-scale secondary ion mass spectrometry [85] could provide powerful ways to sort the targets for downstream whole-genome sequencing and cultivation or study the in situ ecophysiology of not-yet-cultured bacteria in complex environmental samples at the single-cell level [86].

The hybrid assembly method also makes huge contributions to genome resolution of MDM and shows perfect applications on AS according to the examples of Denmark and Hong Kong. It is foreseeable that this technology is a powerful tool for subsequent excavation of genomes especially the ones with high abundance, and can be transferred to other systems like biofilm and DS easily. Genomes will enormously improve our understanding of microbial research. Firstly, genomic information will be conducive to guiding the genome mining even the isolation of crucial microorganisms in WWTPs like anammox [87]. Furthermore, genomes could help to unravel ecological roles of MDM, which is key to predicting ecosystem responses to environmental changes and has a great significance to engineered systems like AS. For example, Patescibacteria might have various host-associated sources due to its reduced metabolic capacities and ultra-small sizes [40, 88], and could influence functional microbes involved in carbon/nitrogen cycles of AS. Besides, an “early warning” system to realize online surveillance of the microbial profile and function prediction of communities by monitoring key strains and the entire microbial community is of great significance [89]. When enough genomes are excavated, the full-length 16S rRNA gene is a perfect messenger between prokaryotic communities and genomes given its wide existence in prokaryotes, sufficient resolution for different taxa, and low requirement of sequencing depth for amplicons. Thus, the more important point is to get HQ or complete genomes that contain full-length 16S rRNA genes from metagenomes, which is difficult to realize by short reads only but perfectly solved by the hybrid assembly. The long reads generated by third-generation sequencing enable the recovery of highly contiguous microbial genomes and short reads could correct insertions and deletions derived from homopolymer regions of long reads sequencing. In fact, the continuous improvement in the sequencing accuracy of long reads will bring another evolution for research. In 2022, the near-finished microbial genomes could be generated from isolates and metagenomes with long reads only by the new sequencing chemistry of Nanopore [90].

Sample selection is of great importance for research. AS is a complex ecosystem with a high diversity of prokaryotes, and sub-samples of AS with lower complexity could further elucidate the genomes that might be omitted in the original communities by hybrid assembly or isolation. Enrichment takes a key role in the transition of the complicated AS consortium to simple systems by removing insignificant interferences and accumulating the abundance of prominent targets. However, the enrichment process is not always smooth sailing because the metabolism of microorganisms is too intricate to make the targets enriched precisely as desired. What is important but usually ignored is that the natural environment is a vast reservoir for prokaryotes that contains numerous and primitive enriched samples, which means the enrichment could be shortened or even skipped if we start from natural samples where the objectives are highly abundant [56]. The distribution of “wanted list” OTUs in other ecosystems of EMP data shows us a good demonstration. What can be predicted is that the cultivation of OTU_68 (g_Romboutsia) might be easier if we attempt to isolate them from animal_digest_gut instead of AS. Of course, the idea of enriching the targets from other environments where they are highly abundant makes sense only when the same 16S rRNA gene and in particular its V4 region is discriminating enough. For some branches of the tree of life, very different strains/species have the same sequence. Getting the sequence of the full-length 16S rRNA gene or the full ribosomal operon may improve the taxa resolution and help to solve this issue.

## Conclusions

This work conducted a worldwide MDM survey in WWTPs using the public database. Results of EMP and WWTPs samples show relatively low genome-sequenced proportions of prokaryotes and the high abundance of MDM in WWTPs. More importantly, a few predominant taxa contribute to high relative abundances in different systems of WWTPs. The 71 OTUs proposed in the “wanted list” and 4 phyla still lack representative genomes. All these results indicate that prokaryotes in WWTPs need further excavation. Future works should put more effort to mine HQ/complete genomes that contain full-length 16S rRNA genes and get isolates for the following ecophysiological research of MDM in WWTPs with multiple methods. These endless efforts will help us eliminate the darkness in the microbial world and bring a bright future to the ecosystem analysis.

## Supplementary Information

**Additional file 1: Figure S1.** The genome number of different phyla in GTDB, including (a) bacteria and (b) archaea. **Figure S2.** The length and quantity distribution of 16S rRNA gene for GTDB R207. **Figure S3.** Genome sequenced results of environments in EMP and worldwide WWTPs. Yellow represents the genome-sequenced proportion of taxa (P_number_) and green represents the sum of corresponding relative abundance (P_abundance_). Amplicons share 100% identity and 100% coverage with the sequenced genomes. For the box plots, the middle line indicates the median, the box represents the 25^th^–75^th^ percentiles, and the error bar indicates the 10^th^–90^th^ percentiles of observations. The results are based on the analysis of 10,000 EMP samples with EMPO level 2 and the worldwide WWTPs samples. **Figure S4.** Genome sequenced results of environments in EMP and worldwide WWTPs with (a) 98.7% and (b) 97% identity. Yellow represents the genome-sequenced proportion of taxa (P_number_) and green represents the sum of corresponding relative abundance (P_abundance_). The results are based on the analysis of 10,000 EMP samples with EMPO level 3 and the worldwide WWTPs samples. **Figure S5.** The correlation between P_abundance_/P_number_ and α-diversity indices of EMP and WWTPs samples, including (a) Observed OTUs, (b) Chao 1, and (c) Shannon. **Figure S6.** The proportions of total abundance and cell number of sequences whose relative abundance <0.1% in WWTPs (AS: activated sludge, DS: digestion sludge). **Figure S7.** The abundance (cell number) of top 10 phyla in AS with Silva 138 database. **Figure S8.** The overlap of sequenced genomes among three types of samples in WWTPs (AS: activated sludge, DS: digestion sludge).

**Additional file 2: Table S1.** List of projects. **Table S2.** The “wanted list” of activated sludge at global level. **Table S3.** The “wanted list” of activated sludge at global level (Strength). **Table S4.** The “wanted list” of activated sludge at global level (loose). **Table S5.** The quantity of ASVs and corresponding aligned genomes in different samples. **Table S6.** The list of sequenced genomes in activated sludge. **Table S7.** The list of sequenced genomes in Biofilm. **Table S8.** The list of sequenced genomes in anaerobic digestion sludge.

## Data Availability

The raw nucleotide sequence data used in this study have been deposited in the NCBI database and the project ID was listed in Table S1 of the supplementary information.

## Abbreviations

WWTPs: Wastewater treatment plants
MAGs: Metagenome-assembled genomes
MDM: Microbial dark matter
GTDB: Genome Taxonomy Database
NCBI: National Center for Biotechnology Information
EMP: Environmental Microbiome Project
AS: Activated sludge
DS: Digestion sludge
ASVs: Amplicon sequence variants
OTUs: Operational taxonomic units
HQ: High-quality
MQ: Medium-quality
LQ: Low-quality
Zi: Within-module connectivity
Pi: Among-module connectivity
EBPR: Enhanced biological phosphorus removal
CRAT: Conditionally rare or abundant taxa
