# Supplementary material for: The microbial dark matter and “wanted list” in worldwide wastewater treatment plants
Source: Microbiome. 2023 Mar 28;11:59. doi: 10.1186/s40168-023-01503-3 (PMC10045942; doi:10.1186/s40168-023-01503-3)
Supplement: Supplementary file 2 — Additional file 1: Figure S1. The genome number of different phyla in GTDB, including (a) bacteria and (b) archaea. Figure S2. The length and quantity distribution of 16S rRNA gene for GTDB R207. Figure S3. Genome sequenced results of environments in EMP and worldwide WWTPs. Yellow represents the genome-sequenced proportion of taxa (Pnumber) and green represents the sum of corresponding relative abundance (Pabundance). Amplicons share 100% identity and 100% coverage with the sequenced genomes. For the box plots, the middle line indicates the median, the box represents the 25th–75th percentiles, and the error bar indicates the 10th–90th percentiles of observations. The results are based on the analysis of 10,000 EMP samples with EMPO level 2 and the worldwide WWTPs samples. Figure S4. Genome sequenced results of environments in EMP and worldwide WWTPs with (a) 98.7% and (b) 97% identity. Yellow represents the genome-sequenced proportion of taxa (Pnumber) and green represents the sum of corresponding relative abundance (Pabundance). The results are based on the analysis of 10,000 EMP samples with EMPO level 3 and the worldwide WWTPs samples. Figure S5. The correlation between Pabundance/Pnumber and α-diversity indices of EMP and WWTPs samples, including (a) Observed OTUs, (b) Chao 1, and (c) Shannon. Figure S6. The proportions of total abundance and cell number of sequences whose relative abundance <0.1% in WWTPs (AS: activated sludge, DS: digestion sludge). Figure S7. The abundance (cell number) of top 10 phyla in AS with Silva 138 database. Figure S8. The overlap of sequenced genomes among three types of samples in WWTPs (AS: activated sludge, DS: digestion sludge). [file 40168_2023_1503_MOESM1_ESM.docx]

**Supplementary Information**

**for**

**The microbial dark matter and “wanted list” in worldwide wastewater treatment plants**

Yulin Zhang^1^, Yulin Wang^1^, Mingxi Tang^1^, Jizhong Zhou^4^, Tong Zhang^1, 2, 3^*

^1^ Environmental Microbiome Engineering and Biotechnology Lab, Department of Civil Engineering, The University of Hong Kong, Pokfulam Road, Hong Kong, China.

^2^ Shenzhen Bay Laboratory, Shenzhen, China

^3^ Peking University Shenzhen Graduate School, Shenzhen, China

^4^ Institute for Environmental Genomics, Department of Microbiology and Plant Biology, and School of Civil Engineering and Environmental Sciences, University of Oklahoma, Norman, OK, USA

*Corresponding author. Phone: +852-28578551. Fax: +852-25595337. E-mail: [zhangt@hku.hk](mailto:zhangt@hku.hk).

# Contents

This file contains 8 supporting figures (Figure S1-S8) in a total of 14 pages.

# The conditions of the 16S rRNA gene in GTDB R207

The 16S rRNA genes from GTDB R207 genomes were extracted by using nhmmer [1] with the 16S rRNA model (RF00177 and RF01959) from the Rfam database [2] with Infernal (version 1.1.4) [3]. Sequences whose lengths were between 300 bp and 1700 bp with the *E*-value <=1e^-6^ were reported. Finally, we got 525,073 16S rRNA reads with the majority of them having the length between 1500 bp to 1600 bp (Figure S2). The 16S rRNA distributed in 256,120 genomes, counting ~80% of the total genomes in GTDB R207.

# The taxonomy distribution of sequenced genomes in WWTPs

The activated sludge (AS) samples were successfully aligned with 7,699 genomes, including 7,597 bacterial genomes and 102 archaeal genomes (Table S6). The 7,597 bacteria genomes were classified into 74 phyla, with the highest top five of Proteobacteria (2,316 genomes), Firmicutes (1,426 genomes), Bacteroidota (1,331 genomes), Actinobacteriota (869 genomes), and Firmicutes_A (859 genomes). The remaining genomes were assigned to other miscellaneous bacterial phyla. For archaea, the 102 genomes were assigned to 8 archaeal phyla with the top three phyla of Halobacteriota (45 genomes), Methanobacteriota (31 with genomes), and Thermoproteota (with 12 genomes). These phyla are all key groups commonly found in AS [4, 5]. The biofilm samples aligned 3,379 genomes (3,345 bacteria and 34 archaea, Table S7). For digestion sludge (DS), 4,830 genomes (4,678 bacteria and 152 archaea, Table S8). Though there are variations in the specific quantities, the top three phyla of bacteria and archaea for biofilm and DS samples are always consistent with the AS samples. There was a large overlap (~50%) of genomes among these three types of samples (Figure S8), at the same time each system also had its independent types of prokaryotes.

Among the taxonomy distribution of sequenced genomes, the top 5 phyla of the three systems, AS, biofilm, and DS, are all key groups commonly in worldwide WWTPs [4, 5]. What’s more, the fact that top phyla are also dominant in GTDB (Figure S1) might identify our suppose above again, i.e. the prokaryotes that have been cultured and suffer low abundance in WWTPs might originate and be isolated from other ecosystems. The three types of samples have a large overlap (~50%) among the genomes is reasonable (Figure S8). In the wastewater treatment process, biofilm systems are combined with AS, such as the moving bed biofilm reactor (MBBR). DS at WWTPs receives substantial amounts of prokaryotes via feed streams in the form of primary sludge or surplus AS periodically [6, 7], which means many of the prokaryotes in AS will transfer to DS though they might be not growing, presumably inactive or dying off there. Kirkegaard et al [8] conducted thirty-two fullscale DS over six years and found the communities of individual digesters surveyed were remarkably similar – with only 300 operational taxonomic units (OTUs) representing 80% of the total reads across all plants, and 15% of these identified as non-growing and possibly inactive immigrating microbes. At the same time, each system also had its independent prokaryotes types with different ecological niches due to the diverse operation parameters and individual functions.

# Potential functions of the sequenced genomes in WWTPs

We applied the MIDAS4 database [9, 10] to identify the potential functions of genomes in WWTPs and found they demonstrate a wide range of abilities in the biogeochemical cycling of carbon (C), nitrogen (N), and phosphorus (P). Plenty of prokaryotes in AS are aerobic heterotrophs and could utilize complex organic compounds, meaning they all make contributions to promoting the C cycle in WWTPs, such as certain Saprospiraceae species [11]. The N cycle mainly involves N oxidation prokaryotes of Nitrospira, Nitrotoga, and Nitrosomonas and denitrifiers like Thauera and Rhodoferax that are all abundant in WWTPs. Besides, the putative polyphosphate accumulating organisms (PAOs), Candidatus Accumulibacter usually showed in enhanced biological P removal (EBPR) systems, and several glycogen accumulating organisms (GAOs) including Candidatus Competibacter and Candidatus Contendobacter also exist. Apart from the taxon mentioned above which are all defined as the core components of WWTPs and make great attributions to the wastewater treatment efficiency, other lineages have obvious influence on the treatment efficiency of activated sludge or are closely related to human health. For example, Zoogloea species, a core denitrifiers genus in AS communities, can enhance the flocculation effect, and certain Saprospiraceae species play an important role in hydrolysis in EBPR systems [12]. In contrast, Actinomycetes are notorious for scum formation; Gordonia could result in sludge foaming; and Neomegalonema, most often observed in industrial plants, is implicated in bulking. Arcobacter species, one of the most abundant genera in influent wastewater, are associated with human and animal diseases [13].


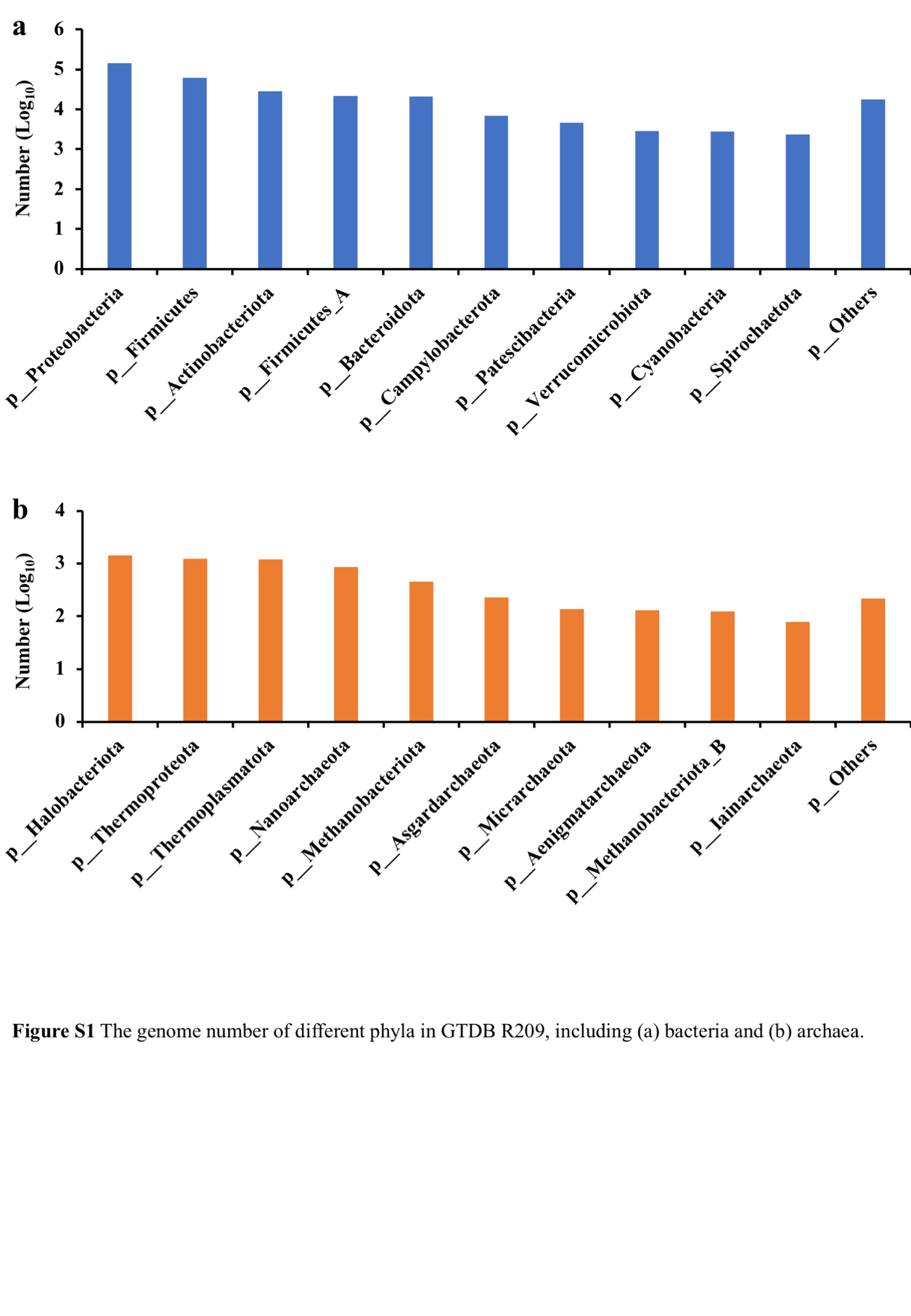


**Figure S1** The genome number of different phyla in GTDB, including (a) bacteria and (b) archaea.


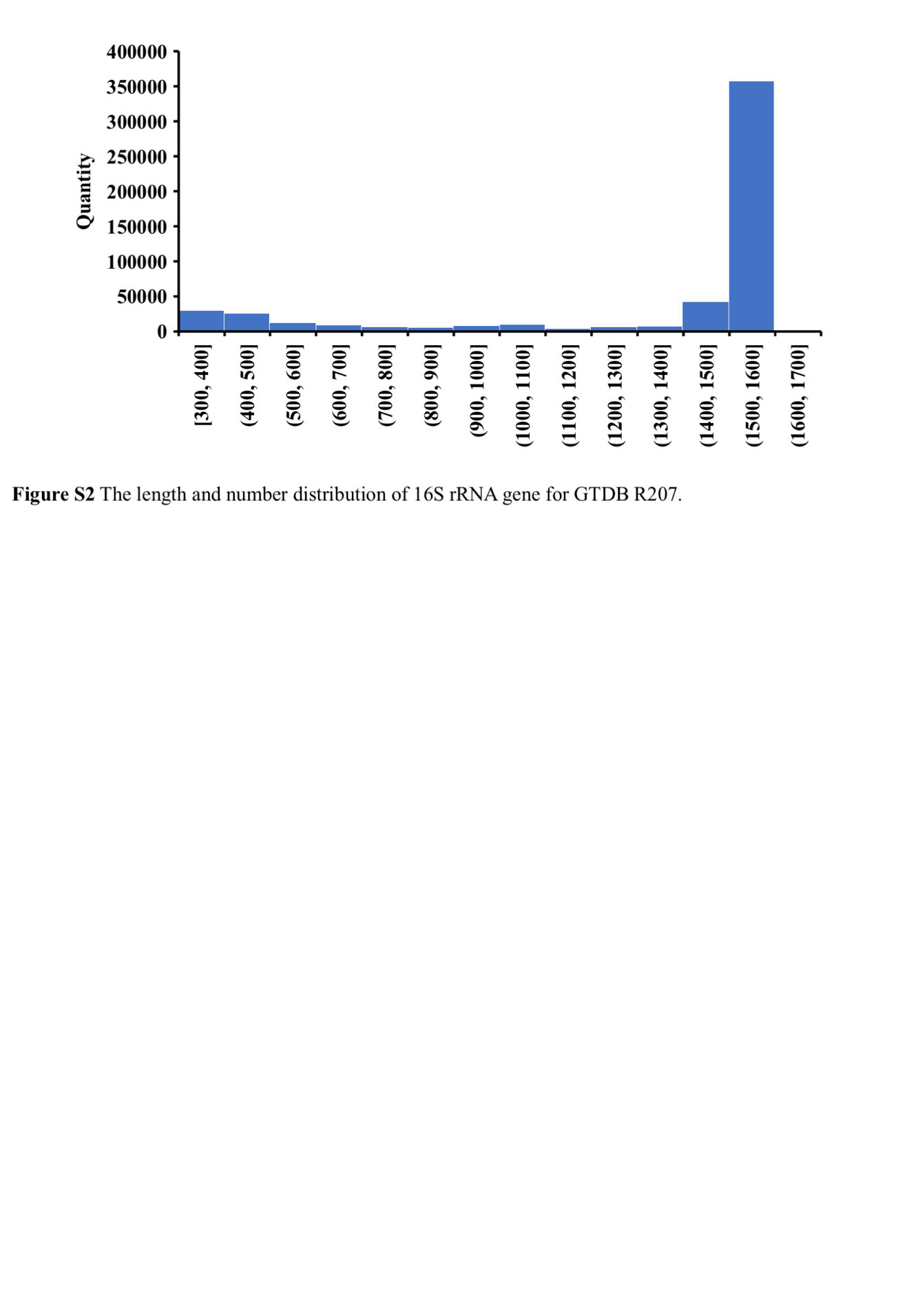


**Figure S2** The length and quantity distribution of 16S rRNA gene for GTDB R207.


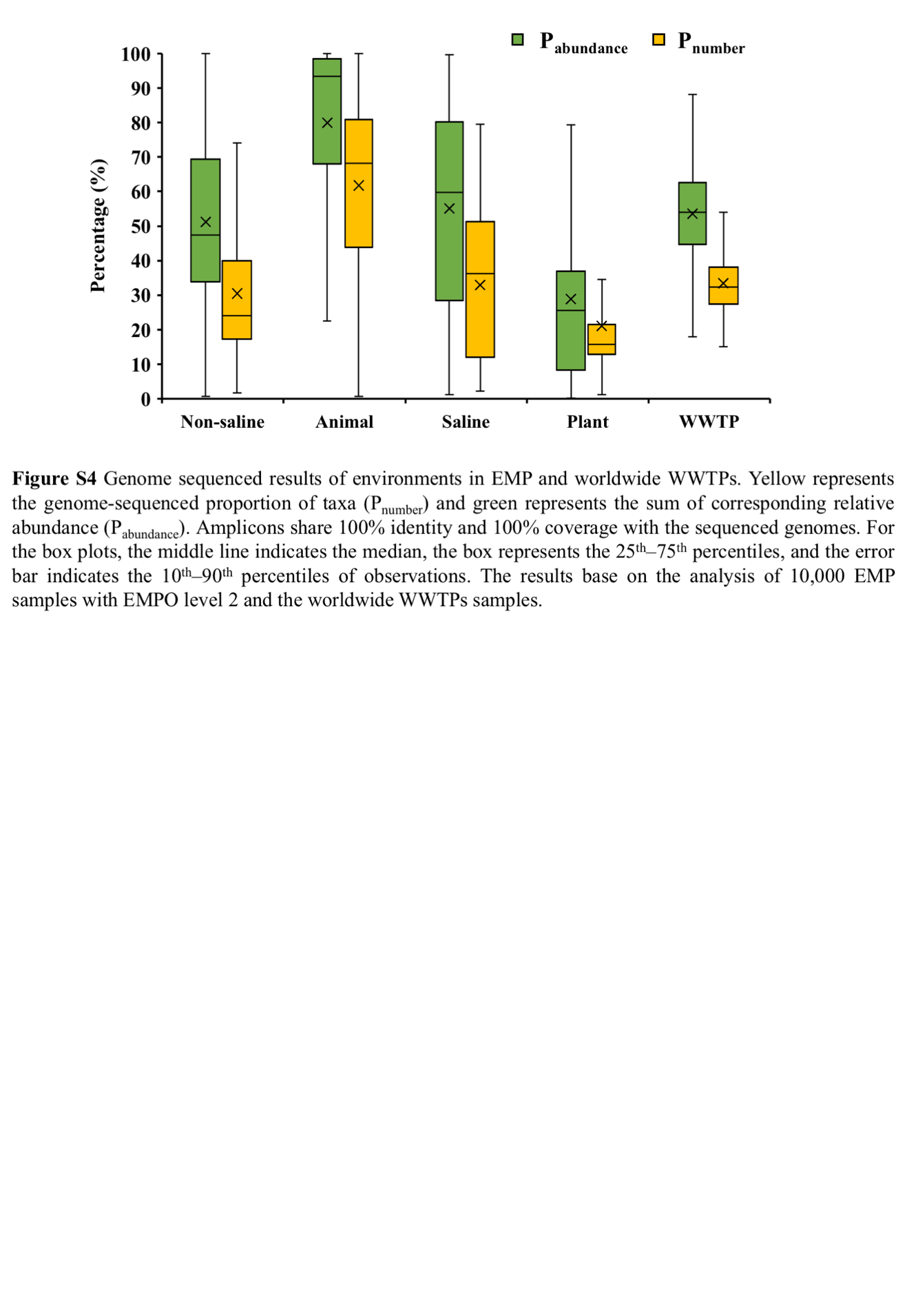


**Figure S3** Genome sequenced results of environments in EMP and worldwide WWTPs. Yellow represents the genome-sequenced proportion of taxa (P_number_) and green represents the sum of corresponding relative abundance (P_abundance_). Amplicons share 100% identity and 100% coverage with the sequenced genomes. For the box plots, the middle line indicates the median, the box represents the 25^th^–75^th^ percentiles, and the error bar indicates the 10^th^–90^th^ percentiles of observations. The results are based on the analysis of 10,000 EMP samples with EMPO level 2 and the worldwide WWTPs samples.


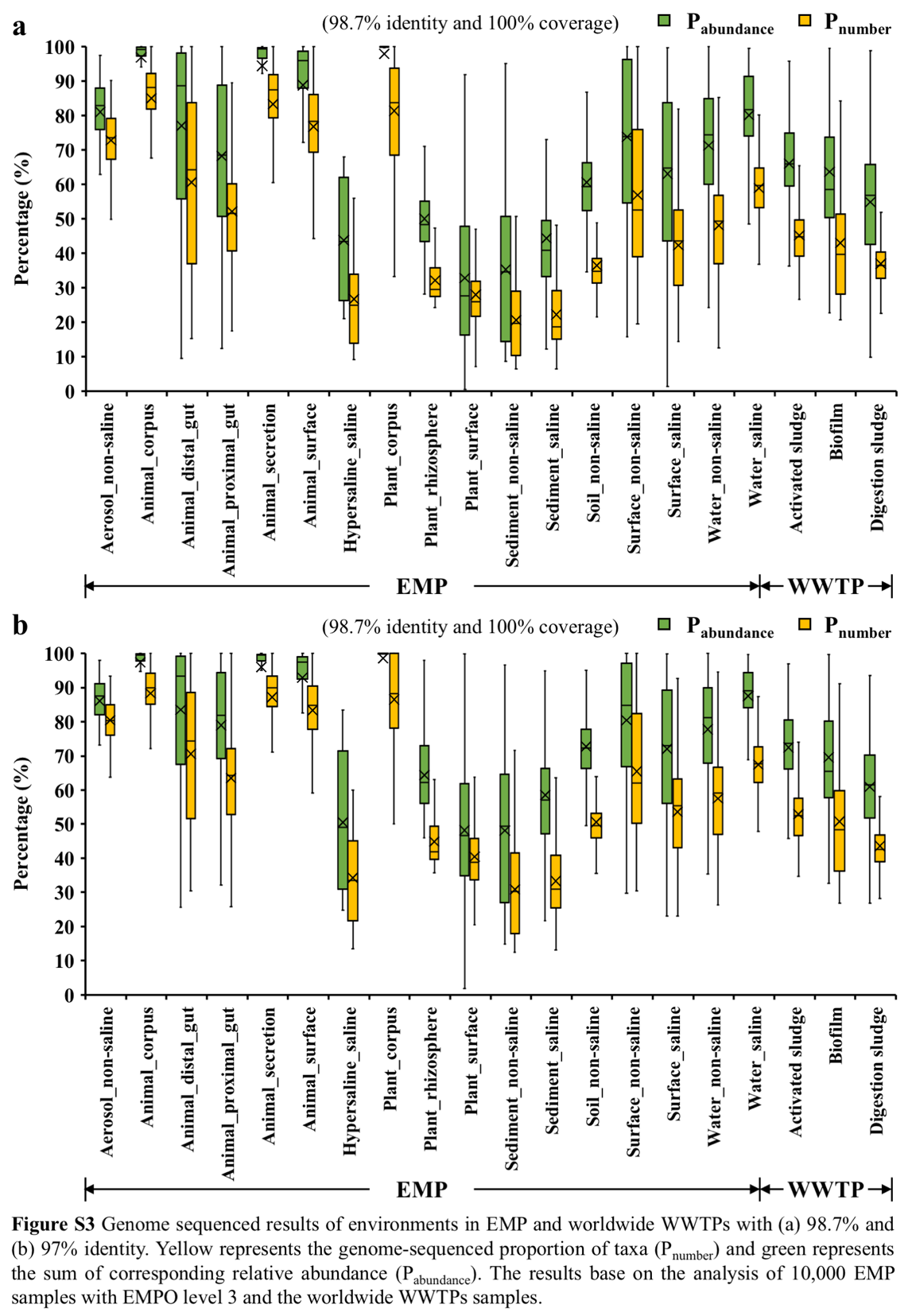


**Figure S4** Genome sequenced results of environments in EMP and worldwide WWTPs with (a) 98.7% and (b) 97% identity. Yellow represents the genome-sequenced proportion of taxa (P_number_) and green represents the sum of corresponding relative abundance (P_abundance_). The results are based on the analysis of 10,000 EMP samples with EMPO level 3 and the worldwide WWTPs samples.


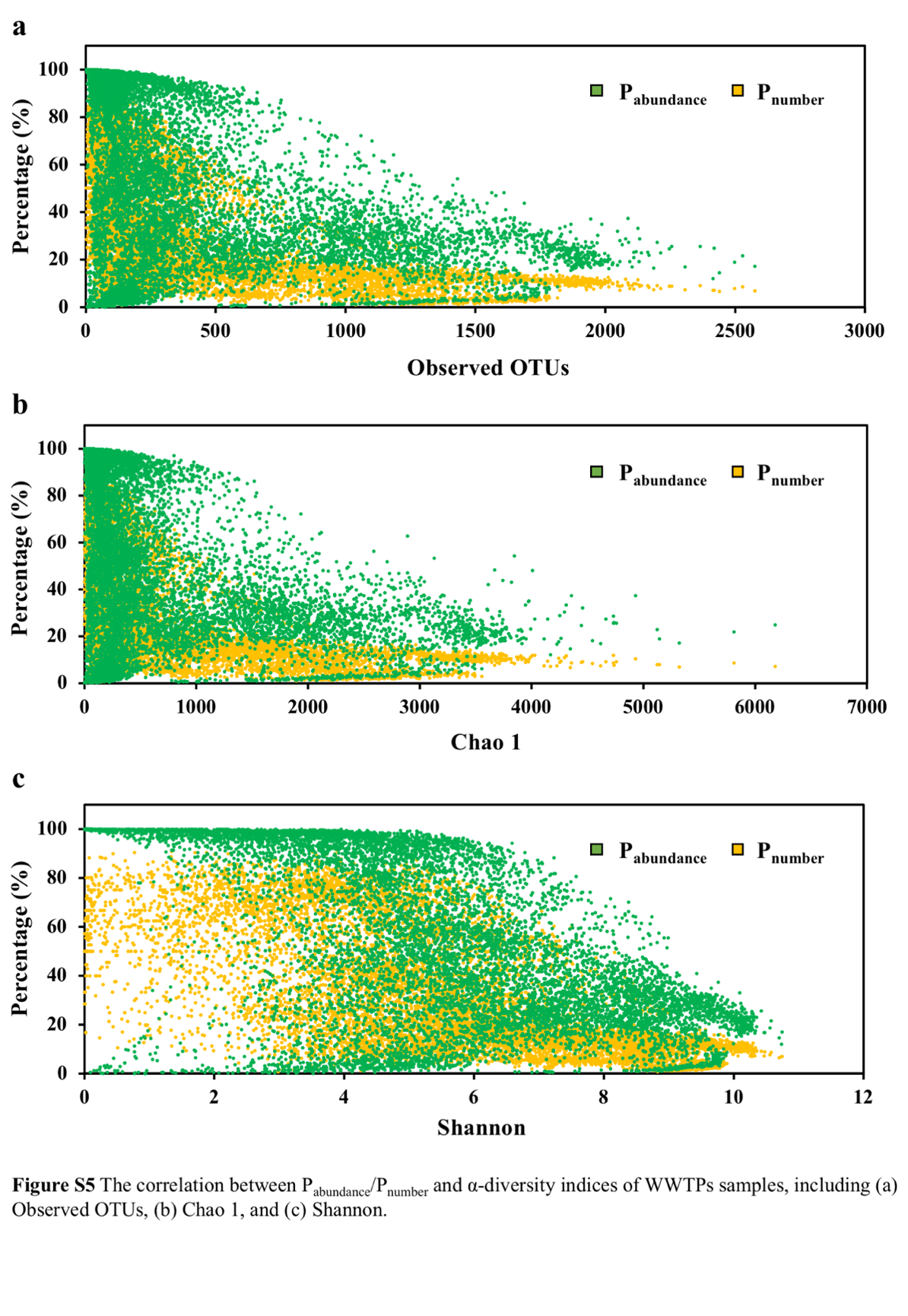


**Figure S5** The correlation between P_abundance_/P_number_ and α-diversity indices of EMP and WWTPs samples, including (a) Observed OTUs, (b) Chao 1, and (c) Shannon.

**
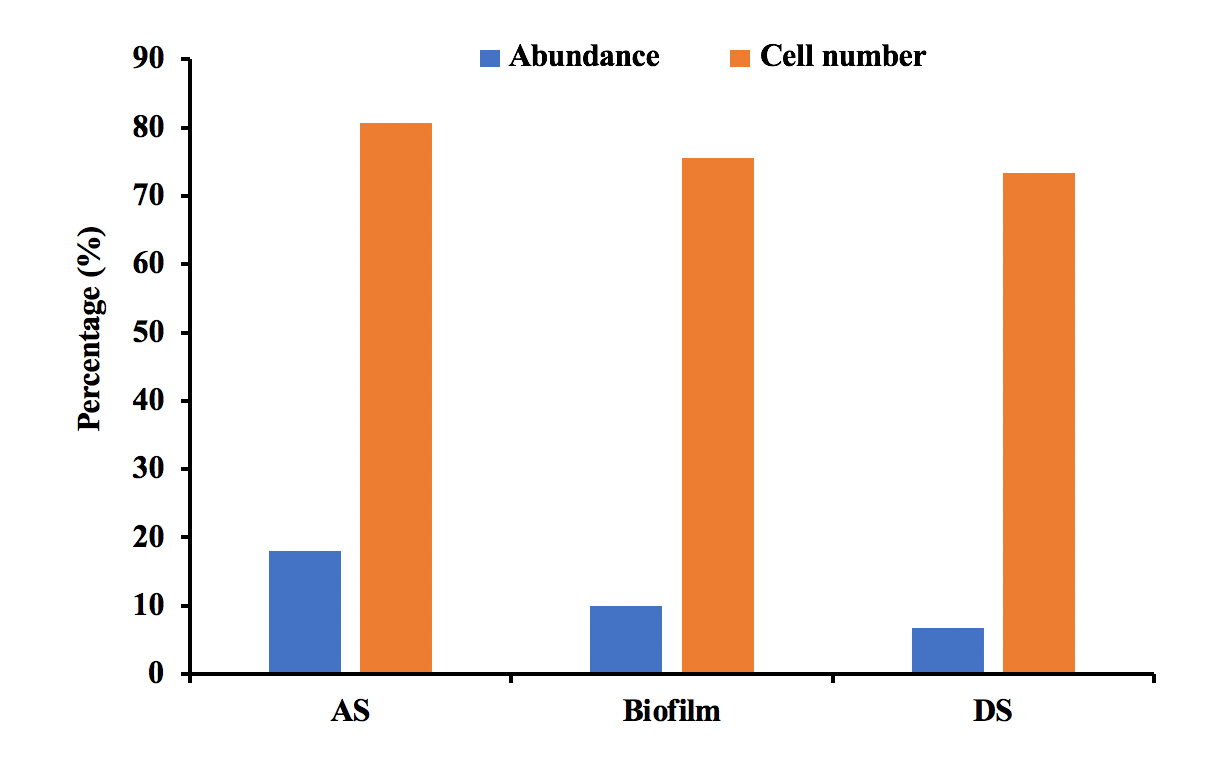
**

**Figure S6** The proportions of total abundance and cell number of sequences whose relative abundance <0.1% in WWTPs (AS: activated sludge, DS: digestion sludge).


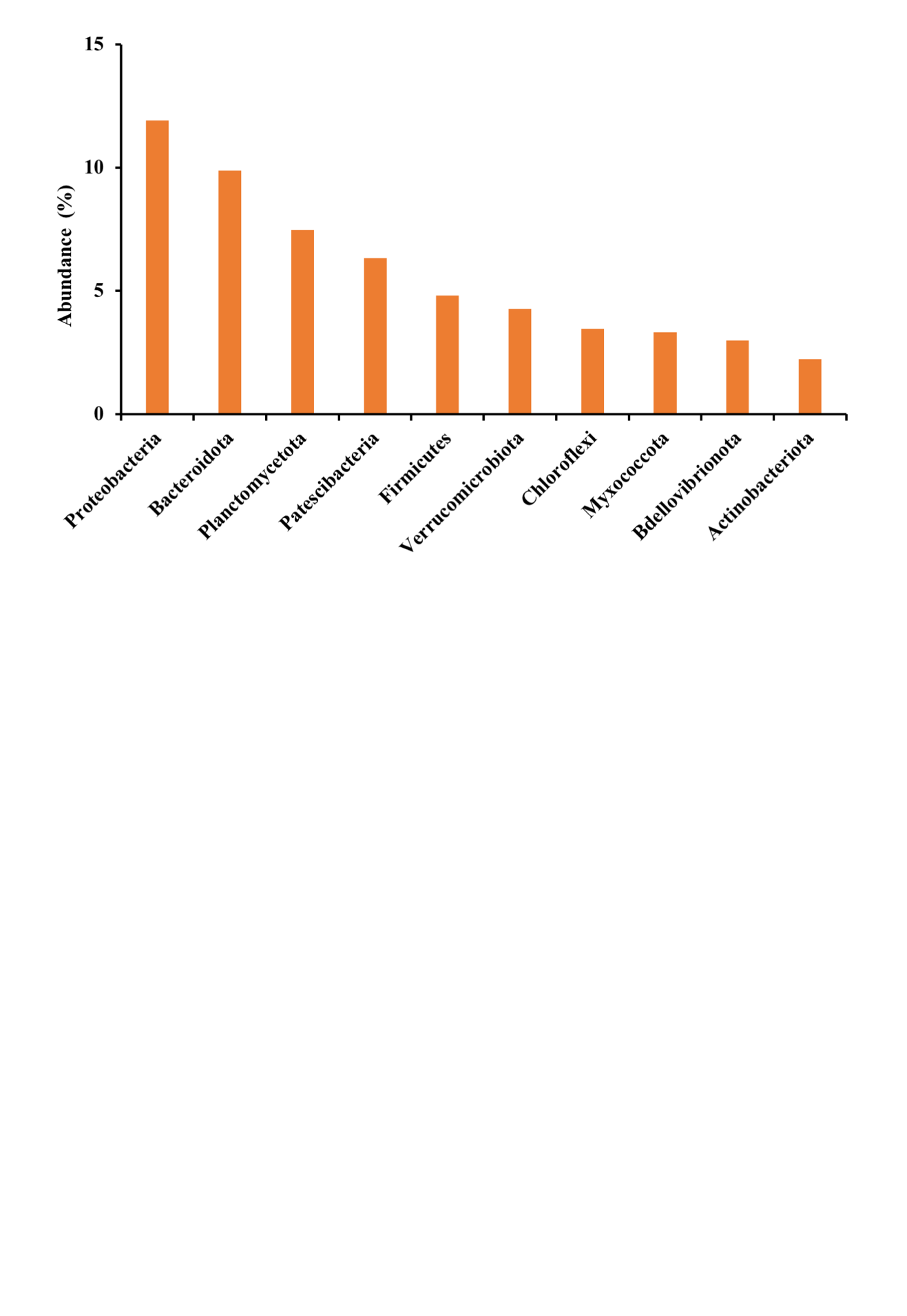


**Figure S7** The abundance (cell number) of top 10 phyla in AS with Silva 138 database.


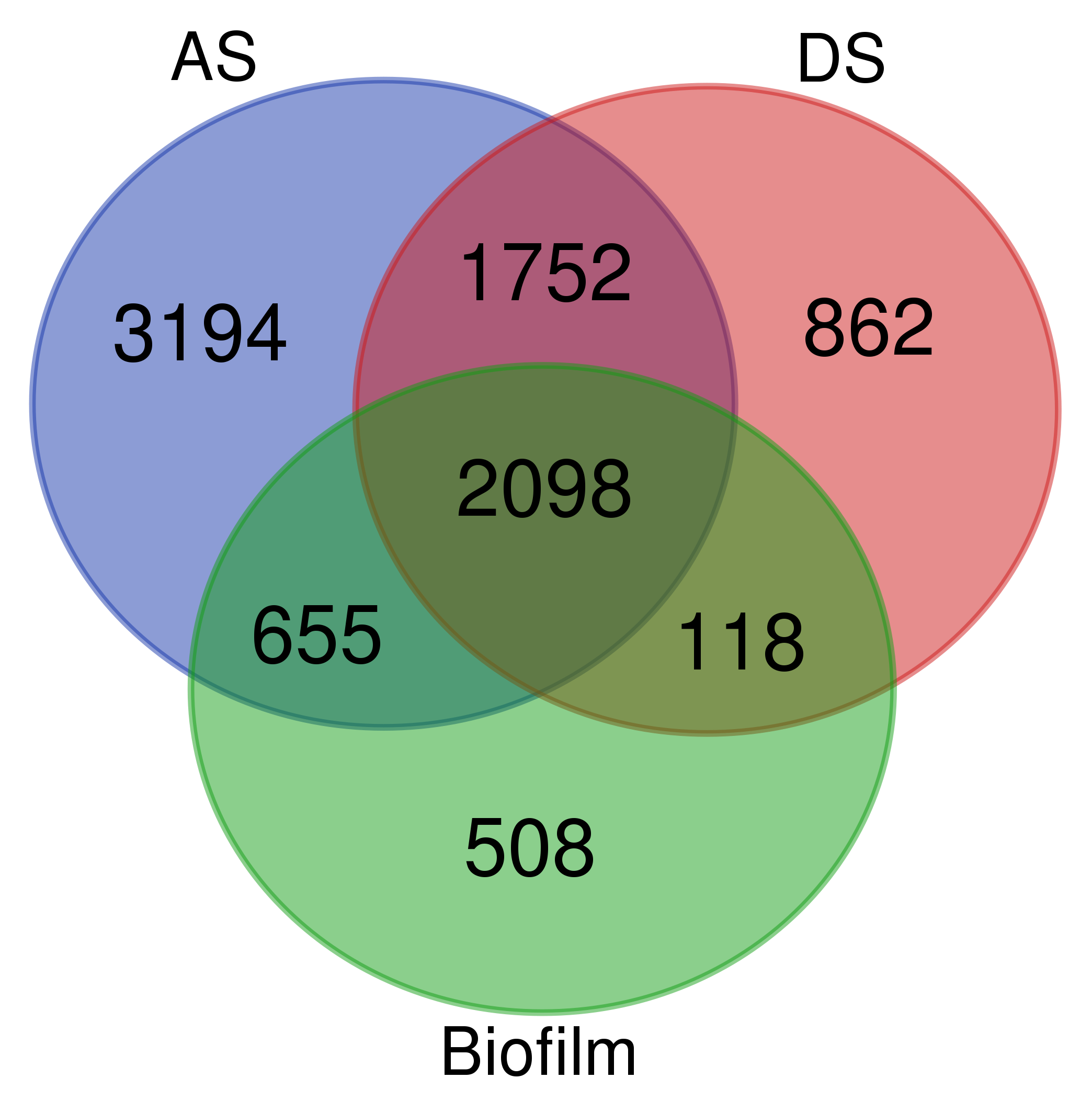


**Figure S8** The overlap of sequenced genomes among three types of samples in WWTPs (AS: activated sludge, DS: digestion sludge).

# References

1. Wheeler TJ, Eddy SR. Nhmmer: DNA homology search with profile hmms. Bioinformatics. 2013;29(19):2487-9.

2. Kalvari I, Nawrocki EP, Ontiveros-Palacios N, Argasinska J, Lamkiewicz K, Marz M, et al. Rfam 14: Expanded coverage of metagenomic, viral and microrna families. Nucleic Acids Res. 2020;49(D1):D192-D200.

3. Nawrocki EP, Eddy SR. Infernal 1.1: 100-fold faster rna homology searches. Bioinformatics. 2013;29(22):2933-5.

4. Ye L, Mei R, Liu W-T, Ren H, Zhang X-X. Machine learning-aided analyses of thousands of draft genomes reveal specific features of activated sludge processes. Microbiome. 2020;8(1):16.

5. Ju F, Zhang T. Bacterial assembly and temporal dynamics in activated sludge of a full-scale municipal wastewater treatment plant. ISME Journal. 2014;9:683.

6. Jiang C, Peces M, Andersen MH, Kucheryavskiy S, Nierychlo M, Yashiro E, et al. Characterizing the growing microorganisms at species level in 46 anaerobic digesters at danish wastewater treatment plants: A six-year survey on microbial community structure and key drivers. Water Res. 2021;193:116871.

7. Ju F, Lau F, Zhang T. Linking microbial community, environmental variables, and methanogenesis in anaerobic biogas digesters of chemically enhanced primary treatment sludge. Environ Sci Technol. 2017;51(7):3982-92.

8. Kirkegaard RH, McIlroy SJ, Kristensen JM, Nierychlo M, Karst SM, Dueholm MS, et al. The impact of immigration on microbial community composition in full-scale anaerobic digesters. Sci Rep. 2017;7(1):9343.

9. Nierychlo M, Andersen KS, Xu Y, Green N, Jiang C, Albertsen M, et al. Midas 3: An ecosystem-specific reference database, taxonomy and knowledge platform for activated sludge and anaerobic digesters reveals species-level microbiome composition of activated sludge. Water Res. 2020;182:115955.

10. Dueholm MKD, Nierychlo M, Andersen KS, Rudkjøbing V, Knutsson S, Arriaga S, et al. Midas 4: A global catalogue of full-length 16s rrna gene sequences and taxonomy for studies of bacterial communities in wastewater treatment plants. Nat Commun. 2022;13(1):1908.

11. Xia Y, Kong Y, Thomsen TR, Nielsen PH. Identification and ecophysiological characterization of epiphytic protein-hydrolyzing saprospiraceae (“candidatus epiflobacter” spp.) in activated sludge. APPL ENVIRON MICROB. 2008;74(7):2229-38.

12. Nielsen PH, Saunders AM, Hansen AA, Larsen P, Nielsen JL. Microbial communities involved in enhanced biological phosphorus removal from wastewater—a model system in environmental biotechnology. Curr Opin Biotechnol. 2012;23(3):452-9.

13. Ferreira S, Queiroz JA, Oleastro M, Domingues FC. Insights in the pathogenesis and resistance of arcobacter: A review. Critical Reviews in Microbiology. 2016;42(3):364-83.
